# Notes and News

**Published:** 1902-07-19

**Authors:** 


### NOTES AND NEWS.

PRINCESS LOUISE OF SCHLESWIG-HOLSTEIN will open a bazaar at the Ascot Cottage Hospital, on Saturday the 19th.

MR. PALMER, of Reading, has presented £2,000 to the Royal Berkshire Hospital.

A CASE of plague has been reported as under treatment at the principal Jewish hospital at Odessa. Great secrecy is maintained, but the medical plague committee who worked during last year's outbreak has been reappointed.

THE number of candidates—between seventy and eighty—who have presented themselves for selection at the forthcoming examination of the Royal Army Medical Corps, shows that the service has now become popular. There are only thirty vacancies.

THE Metropolitan Asylums Board had the subject of consumption under consideration last week. It was reported that the Lambeth Borough Council had instituted a system of voluntary notification of consumption. The Hackney Borough Council urged the establishment of a sanatorium, and it was decided to issue a general reply to the question which had been raised in a number of districts.

WHEN strikes are carried to their logical conclusion they evidently involve the application of the boycott; and the Americans are a logical people. Hence it is in the United States that we find the most complete examples of the cruelty of industrial warfare. It is stated that in the strike of coal miners in Pennsylvania not only are hostile demonstrations being made before the houses of non-union workmen, but medical practitioners are being warned, under pain of severe punishment, not to attend any of the sick or injured among the men at work in the mines. What about the neutrality of the Red Cross? We have heard much about the savagery of war, but war as we have recently seen it is as the milk of human kindness compared with the savagery displayed in a really well fought out strike.

SMALL-POX has assumed a serious form in Swansea. The number of cases in the Borough Hospital amounts to upwards of thirty-six. The Swansea Summer Assizes will probably be held at Cardiff, and the War Office has been approached with a view to prevent Swansea volunteers from going into camp on Salisbury Plain this year.

IN a lecture at the Trocadero Restaurant last week Mr. J. Patterson, President of the National Cash Register Company, Ohio, eloquently testified to the improved working powers of employees where the employer concerned himself with their physical comfort. He depicted a condition of affairs in his own company which must cause envy to workers in England.

So little interest do the Fellows of the Royal College of Surgeons take in the management of the institution over which they claim the entire control, to the exclusion of the members, that at the annual meeting of the Fellows summoned for July 3rd only five attended, and as a quorum of 30 is required no meeting was held. This is the sixth occasion on which no quorum has been present!

MRS. GARRETT ANDERSON, M.D., presided at the annual prize distribution of the London School of Medicine for Women last week. She mentioned that the most important event of the year's work was the appointment of medical women to fill two resident posts at the Royal Free Hospital. She also enumerated the principal posts gained elsewhere by past students of the college, and reported that one had gained a gold medal from the London University.

IT has been decided to hold a Coronation fête at Earl's Court on July 26th in aid of the hospitals and as a thankoffering for the King's recovery. The Duke of Argyll is honorary president of the Ground Committee, the Lord Mayor vice-president, and Lord Aberdare chairman. The entertainments at Earl's Court will be enlarged considerably. It is intended to send a congratulatory telegram to the King during the fête, representing the feeling of those present.

THE circular letter sent to their collectors for the Hospital Saturday Fund opens a difficult question. The letter states that individual institutions are sending appeals for money to be sent direct to them, instead of to the collectors in various firms contributing to the Fund. The Fund appeals to the collectors not to let the claims of individual institutions interfere with the contribution to institutions in general through the Saturday Fund. We should hardly think that such appeals would have any weight within the organisation of the Fund, as through its medium donors are spared the difficult responsibility of investigating the merits of the institutions, are spared the trouble of distribution themselves, and moreover have the advantage of a large choice of institutions to seek benefit from when necessary. The Saturday Fund does not wish to check the independent effort of any institution to secure support, but the workshop collection is essentially its own, and it is a mistake for hospital managers to interfere with the Fund's particular field of action.

